# Evidence for Involvement of Th17 Type Responses in Post Kala Azar Dermal Leishmaniasis (PKDL)

**DOI:** 10.1371/journal.pntd.0001703

**Published:** 2012-06-19

**Authors:** Gajendra Kumar Katara, Nasim Akhtar Ansari, Avninder Singh, V. Ramesh, Poonam Salotra

**Affiliations:** 1 National Institute of Pathology (ICMR), Safdarjung Hospital Campus, New Delhi, India; 2 Department of Dermatology, Safdarjung Hospital, New Delhi, India; National Institutes of Health, United States of America

## Abstract

**Background:**

Post kala-azar dermal leishmaniasis (PKDL), a dermal sequel of visceral leishmaniasis, caused by *Leishmania donovani*, constitutes an important reservoir for the parasite. Parallel functioning of counter acting immune responses (Th1/Th2) reflects a complex immunological scenario, suggesting the involvement of additional regulatory molecules in the disease pathogenesis.

**Methodology/Principal Findings:**

In the present study, human cytokine/chemokine/receptor specific cDNA array technique was employed to identify modulations in gene expression of host immuno-determinants during PKDL, followed by evaluation of Th17 type responses by analyzing mRNA and protein expression of Th17 markers (IL-23, IL-17, RORγt) and performing functional assays using *Leishmania* antigen (TSLA) or recombinant (rec)IL-17. Array analysis identified key immuno-regulatory molecules including cytokines (TNF-α, IFN-γ, IL-10, IL-17), chemokines (MCP-1, MIP-1α), apoptotic molecules (FasL, TRAIL, IRF-1) and receptors (CD40, Fas). Up regulation in lesional expression of Th17 markers was observed during PKDL compared to control (IL-17 and IL-23, *P = 0.0008*; RORγt, *P = 0.02*). In follow-up samples, chemotherapy significantly down regulated expression of all markers. In addition, lesional expression of IL-17 was confirmed at protein level by Immuno-histochemistry. Further, systemic presence of Th17 responses (IL-17 and IL-23) was observed in plasma samples from PKDL patients. In functional assays, TSLA stimulated the secretion of IL-17 and IL-23 from PBMCs of PKDL patients, while recIL-17 enhanced the production of TNF-α as well as nitric oxide (NO) in PKDL compared to control (TNF-α, *P = 0.0002*; NO, *P = 0.0013*). Further, a positive correlation was evident between lesional mRNA expression of IL-17 and TNF-α during PKDL.

**Conclusion/Significance:**

The results highlight key immune modulators in PKDL and provide evidence for the involvement of Th17 type responses in the disease pathogenesis.

## Author Summary

Post kala azar dermal leishamniasis (PKDL), an unusual dermatosis, develops in 5–15% of apparently cured visceral leishmaniasis cases in India and in about 60% of cases in Sudan. PKDL cases assume importance since they constitute an important human reservoir for the parasite. Host immunological responses, considered as major factors in PKDL development, are poorly understood. Limited studies have been performed to explore the host immune responses and that too, restricted to a few immune parameters. The present study employed cDNA array technique that identified various host immuno-determinants including cytokines, chemokines, apoptotic and signaling molecules which were not reported previously in PKDL. In addition, we showed for the first time that Th17 responses are present during *L. donovani* infection in PKDL which possibly contributes significantly to disease pathogenesis by inducing TNF-α and nitric oxide production. Our findings lead to improved understanding of the host parasite interaction in terms of immune responses and pathology in tissue lesions of PKDL.

## Introduction

Visceral leishmaniasis (VL) or kala azar (KA), a potentially fatal protozoan disease caused by the members of *Leishmania donovani* complex, is endemic in 62 countries; with 200 million people at risk and an estimated 500,000 new cases worldwide annually [1], [2]. A small percentage of apparently cured VL patients and occasionally, persons from endemic areas without a history of VL, develop a dermal manifestation known as post kala-azar dermal leishmaniasis (PKDL), characterized by macular, papular and/or nodular rash. The disease is relatively common in the Indian subcontinent (India, Nepal, and Bangladesh) and East Africa where the causative agent is *L. donovani*. However, the incidence of PKDL varies from 5–15% in India and to 50–60% in Sudan, the reasons for which are presently unknown [3]. PKDL assumes importance being the anthroponotic reservoir of VL in the Indian subcontinent, and contributing to increasing drug resistance [4], [5].

While it is generally accepted that infection with different species of *Leishmania* leads to the establishment of different clinical forms, the same species of this parasite also leads to different disease manifestation in VL and PKDL demonstrating that the host's immune responses plays a vital role in the disease pathogenesis. Various factors implicated in the development of PKDL include nutrition, genetics, inadequate treatment of VL and immune suppression or reinfection [3]. Studies have suggested PKDL as a drug dependent manifestation since it is reported more frequently in SAG treated VL patients [6], [7]. However, cases of PKDL develop even after treatment with other antileishmanial drugs [8].

A major role of immune responses in the development of PKDL is well recognized [9], [10]. Antecedently, simultaneous presence of both Th1/Th2 responses with increased ratio of TNF-α/IL-10 and involvement of matrix metalloproteinases (MMPs) and tissue inhibitors of MMPs (TIMPs) was documented in tissue lesions of PKDL patients [11], [12]. Further, recent reports documented the presence of T regulatory (Tregs) cells and demonstrated their role in parasite persistence by establishing positive correlation with parasite load in PKDL tissue lesions [13], [14].

Th17 cells represent a newly described T-cell subset, characterized by production of IL-17 [15] and require IL-23 for differentiation and maintenance [16]. Th17 cells play a pivotal role in autoimmunity and chronic inflammatory diseases [17] and participate in defense mechanisms against certain pathogens including *L. donovani* [18], [19]. Further, IL-17 was associated with intensity of infiltration and pathogenesis of cutaneous leishmaniasis (CL) [20].

In PKDL, majority of the studies in the past have typically focused on a small subset of genes as suspected immuno-determinants for understanding the immune regulation. In this study, cDNA array technology was employed to obtain a comprehensive picture of immunological scenario inside lesion tissue during active disease. Further, the elevated expression of IL-17 in gene array and clues from studies on CL incited us to investigate Th17 type responses and their role in PKDL. Here, we show that IL-17 production was enhanced during PKDL and contributed to elevated levels of TNF-α and nitric oxide (NO).

## Materials and Methods

### Patients

PKDL patients originating from Bihar, reporting to the Department of Dermatology, Safdarjung Hospital, New Delhi were included in this study (Table 1). Slit-skin smears stained with Giemsa and histopathology of lesion biopsy for detection of LD bodies was performed for diagnosis of PKDL. In addition, QPCR was used to demonstrate *Leishmania* infection in lesions [21]. HIV positive patients were excluded from this study. Patients were treated with oral Miltefosine (150 mg/day) for 2 months which gave apparent clinical cure in all patients. The healthy individuals, all male, included in the study were from non-endemic area with age range of 18–33 years.

**Table 1:** Major characteristics of the study population.

Patients Characteristics	PKDL (n = 25)
**Age (years) range, (mean±SD)**	17–39, (25.24±6.01)
**Sex (M/F)**	19/6
**Cases reporting history of KA**	21
**History of KA, range in years, (mean±SD)**	2–27, (8.90±6.24)
**Type of PKDL lesions**	
Nodular	11
Macular/Papular	11
Polymorphic	3

Abbreviations: M = Male, F = Female, PKDL = Post kala-azar dermal leishmaniasis, KA = Kala azar.

### Sample collection

Skin biopsies (using 4 mm biopsy punch) from tissue lesions of PKDL patients were collected for RNA isolation in RNA later (Ambion, Austin, TX) and in neutralized formalin for IHC. Biopsies were collected from face or shoulder region. Follow-up samples were collected from the same site as at pre treatment stage, one month after completion of treatment. Heparinized blood was collected for plasma and PBMCs isolation. Normal skin tissues (n = 6, from the shoulder region) and blood (n = 10) were collected from healthy individuals.

### Ethics statement

The study was approved by and carried out under the guidelines of the Ethical Committee of the Safdarjung Hospital, New Delhi, India. All patients and healthy individuals provided written informed consent for the collection of samples and subsequent analysis.

### Analysis of mRNA expression using cDNA arrays

Total RNA was isolated from punch biopsy samples collected from PKDL (n = 6) patients and healthy individuals (n = 6) using Trizol (Invitrogen, Green island, NY) method. RNA samples were pooled in equal amount from each individual. Six micrograms of DNA-free RNA from each group was reverse transcribed, in the presence of 50 µCi of α-^33^P dATP (specific activity ≥2000 Ci/mmol; (Perkin Elmer, San Jose, CA) and gene specific primers for each gene represented on the array. The cDNA microarray (AtlasTM; CLONTECH, Palo Alto, CA) consisted of nylon membranes, spotted with 268 different human genes including those encoding cytokines, chemokines, growth factors, and cellular receptors (http://www.clontech.com/support/tools.asp). Briefly, [^33^P] dATP-labelled cDNA was column purified and hybridized, at high stringency, to cDNA array overnight at 68°C. Membranes were washed at high stringency and exposed overnight to phosphor screens. Image was captured with phosphorImager Typhoon 9210 and analyzed by Imagequant TL software (Amersham Biosciences, Pittsburgh, PA). The intensity of each spot was corrected for background levels and normalized using the housekeeping genes. Array data was analyzed by summing the duplicated intensity signals for each gene in individual experiments and taking the average of 3 technical replicates. The data was expressed as the ratio of mRNA levels in PKDL and controls.

### Quantitative mRNA analysis

Real-time PCR was performed as described earlier [14], using cDNA specific FAM-MGB–labeled Taqman primer sets (Applied Biosystems, Foster City, CA) for IFN-γ (Hs00174143_m1), TNF-α (Hs00174128_m1), IL-10 (Hs00174086_m1), IL-6 (Hs00174131_m1), IL-17(Hs00174086_m1), IL-23 (Hs00166229_m1), IL-12β (Hs01011518_m1), RoRγt (Hs00175480_m1), MCP-1 (Hs00234140_m1), IRF-1(Hs00971965_m1) and CD40 (Hs00386848_m1). VIC-MGB labeled 18S rRNA (4319413E) was used as endogenous control. The relative quantification of products was determined by the number of cycles over endogenous control required to detect the expression of gene of interest.

### Cytokine measurement using ELISA

Cytokine levels of IL-17, IL-23 and TNF-α in plasma and supernatants were determined by ELISA (e-Biosciences, San Diego, CA) in accordance with manufacturer's instructions. The values were calculated as the concentrations in the stimulated cultures minus the unstimulated controls.

### Immunohistochemistry

Skin punch biopsy was collected in neutralized formalin. The tissue was paraffin embedded and 5 µm sections were prepared on polylysine coated glass slides from all skin specimens. Immunohistochemical staining using anti-human IL-17 (Santa-Cruz, San Diego, CA) was performed as described earlier [14]. The immunohistochemical staining for IL-17 was evaluated semi quantitatively by counting the percentage distribution of lymphocytes in the dermal infiltrate showing cytoplasmic staining by counting at 400× magnification. It was scored as 0: no staining or less <10% cells labeled; 1: 11–25% of the cells labeled; 2: 25–50% of the cells labeled; 3: >50% cells positively labeled. Only cytoplasmic staining was considered positive.

### PBMCs isolation, stimulation and cytokine production

PBMCs were isolated from PKDL patients at pre-treatment, post-treatment stage and healthy individuals by density gradient centrifugation with Ficoll-Hypaque (Sigma-Aldrich, St Louis, MO). The cells were cultured in RPMI 1640 (Sigma-Aldrich), 10% FCS (Life Technologies, NY), glutamine, HEPES and antibiotics. Briefly, 5×10^5^ cells were plated in 96 well flat bottom tissue culture plates (Axygen, Union city, CA, USA) and were kept only with media (unstimulated) or stimulated with 10 µg/mL of Phytohemagglutinin (PHA) (Sigma-Aldrich) as a positive control or with total soluble *Leishmania* antigen (TSLA) (10 µg/mL) or rec IL-17 (50 ng/ml) (Peprotech, Rocky Hill, NJ). After 72 hrs of incubation at 37°C and 5% CO_2_, supernatants were collected and stored at −70°C until further analysis.

### Estimation of NO production

Nitrite accumulation, an indicator of NO production, was measured in cell culture supernatants using Griess reagent [22]. Briefly, 50 µl samples of culture supernatants were mixed with an equal volume of Griess reagent (Sigma-Aldrich) and incubated at room temperature for 15 min. The absorbance at 570 nm was measured in a microplate reader. The quantity of the respective nitrite was calculated in ng/ml using NaNO_2_ standard curve.

### Statistical analysis

Statistical analysis was performed with Mann-Whitney test/paired t-test using Graph Pad Prism 5 (GraphPad Software, Inc., San Diego, CA). Correlation was evaluated using Spearman's rank correlation test. *P* values≤0.05 were considered significant.

## Results

### Study subjects

Of the 25 PKDL patients, Leishman-Donovan (LD) bodies were seen in 14 patients and histopathological features were compatible with PKDL in all patients revealing cellular infiltrate consisting of lymphocytes, plasma cells, and macrophages. All samples were positive in *Leishmania* specific quantitative real time PCR (QPCR). In post-treatment cases, subsidence of indurated lesions leaving normal or wrinkled skin, and histopathological absence of disease activity was considered as cured. Further no parasites were detectable by QPCR in any of post-treatment cases.

### Gene expression profile in PKDL

**Figure 1:**
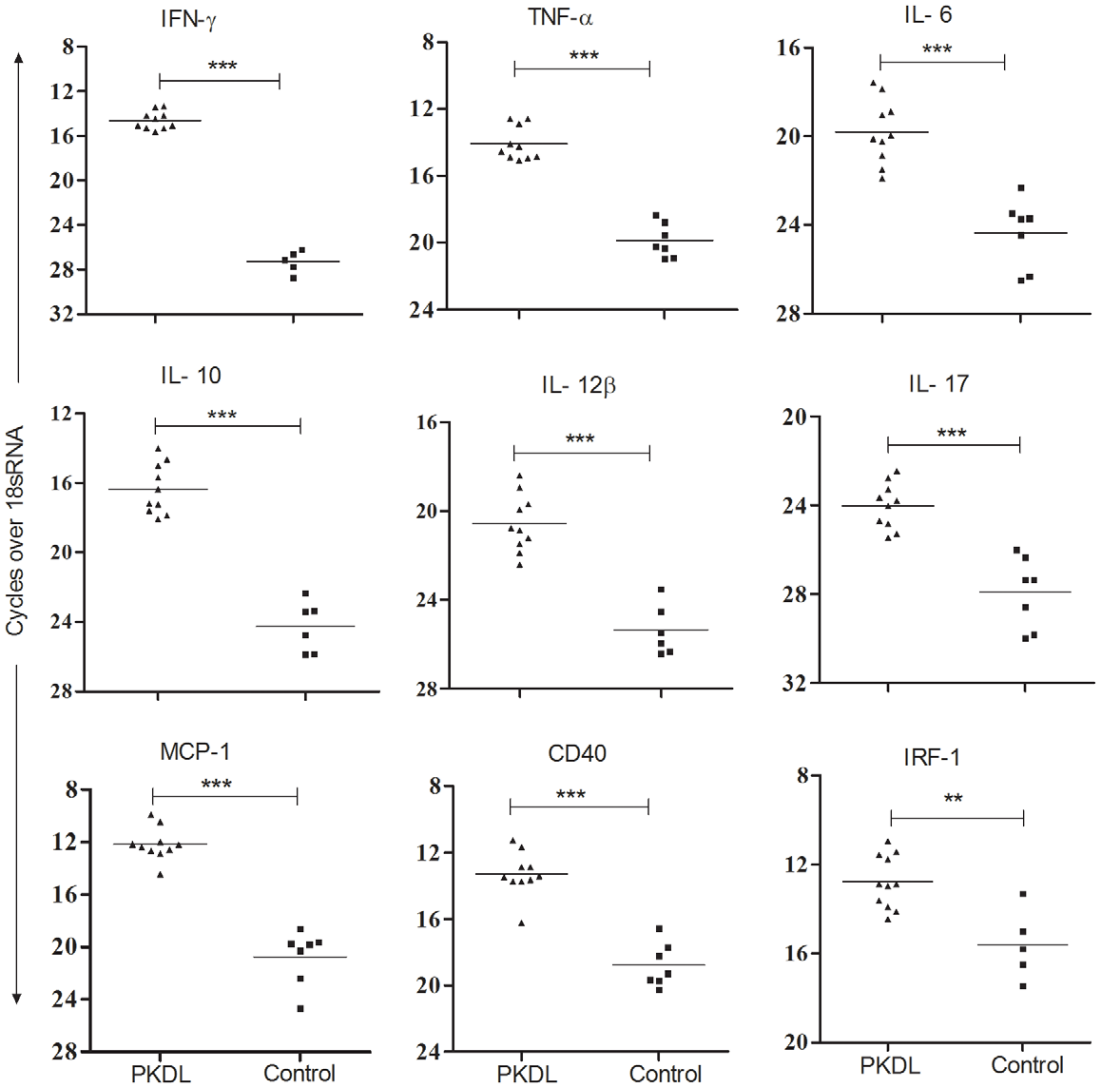
Validation of cDNA array results using real time PCR in tissue lesions of PKDL patients. Relative mRNA levels of IFN-γ, TNF-α, IL-6, IL-10, IL-12β, IL-17, MCP-1, CD40 and IRF-1 was determined by QPCR in tissues lesions of PKDL patients (n = 10) or control tissues (n = 7). The relative quantification of products was determined by the number of cycles over endogenous control (18sRNA) required to detect the gene expression of interest. **P<0.01, and ***P<0.001.

Gene expression analysis using pooled RNA from dermal lesion tissues of PKDL and normal dermal controls was carried out using cDNA arrays. Sixty two genes out of 268 arrayed genes (23.1%) including cytokines, chemokines, receptors and other regulatory molecules, showed modulation 2 fold or more in tissue lesions compared to control. Table 2 identifies selected important genes showing altered expression during PKDL. Genes that were implicated in *Leishmania* pathology (VL, CL, PKDL and MCL), and those related to Th17 type responses were included in the table. Further, the results of gene array were validated in individual PKDL samples by QPCR for selected genes including IFN-γ, TNF-α, IL-10, IL-6, IL-12 β, IL-17, MCP-1, IRF-1 and CD40, all of which showed significantly higher expression in comparison with controls. Out of 7 control samples, mRNA level of IFN-γ and IRF-1was not detectable in two, while IL-10 and IL-12β level was not detectable in one sample (Figure 1).

**Table 2:** Genes showing altered expression in tissue lesions of PKDL (P) compared to human normal skin (HC).

Gene Name	Gene Accession Number	Major Functions	Relative mRNA expression (P/HC)
**Cytokine & Chemokine**			
Tumor necrosis factor-α (TNF-α)	X01394	Proinflammatory cytokine, cell proliferation, differentiation, apoptosis and coagulation	9.0
C-chemokine ligand (CCL)-2 (MCP-1)	M24545	Chemokine activity, immunoregulatory and inflammatory processes	7.3
IL(interlukin)-1β	K02770	Mediator of the inflammatory response, cell proliferation, differentiation, and apoptosis	5.1
IL-17	U32659	Proinflammatory cytokine, high levels are associated with several chronic inflammatory diseases	5.0
CCL3 (MIP-1α)	M23452	Inflammatory responses	4.8
IL-10	M57627	Pleiotropic effects in immunoregulation and inflammation	3.8
IL-12β	M65290	Acts on T and natural killer cells, mediate long-term protection to an intracellular pathogen	3.0
Transforming growth factor (TGF)-β	J03241	Embryogenesis and cell differentiation	2.9
CXCL2	X53799	Chemokine with inflammatory activity	2.9
IL-6	X04602	Functions in inflammation and the maturation of B cells	2.7
IL-4	M13982	Pleiotropic cytokine, regulator of NO synthesis	2.6
IL-8	Y00787	Chemoattractant and inflammatory response	2.5
Interferon (IFN)-γ	X01992	Immunoregulatory and anti-tumor properties, activator of macrophages	2.4
IK cytokine	X01992	Down regulator of HLA class II	2.2
IL-2	A14844	Proliferation of T and B lymphocytes	2.2
CCL5	M21121	Chemoattractant for monocytes, T cells and eosinophils	0.47
**Receptors**			
IL-2receptor-γ	D11086	Signaling initiation and T cell proliferation	6.0
TRAILR2	AF016268	Apoptosis mediation	3.9
CD40	X60592	T cell-dependent immunoglobulin class switching, memory B cell development	3.7
C-chemokine receptor-1	D10925	Receptor for CCL3, chemokine mediated signaling	3.4
IL-4 receptor	X52425	Receptor for IL-4, promote differentiation of Th2 cells	2.8
Fas	Z70519	physiological regulation of programmed cell death	2.2
IL-1receptor type II	X59770	Acts as a decoy receptor for its ligands	0.45
**Others**			
IFN regulatory factor-1 (IRF-1)	X14454	Acts as an activator of interferon-α and β transcription	8.0
FASL	D38122	Ligand for Fas, triggering of apoptosis	4.1
Epstine bar virus-3 (EBI-3)	L08187	IL-27 formation for activation of JAK/STAT pathway	3.4
CD40 ligand	L07414	Ligand for CD40, regulates B cell function	3.3
Protein tyrosine kinase-7	U33635	Tyrosine kinase, cell adhesion molecule	2.6
TRAIL	U57059	Induces apoptosis in transformed and tumor cells	2.2
Programmed cell death 1	AF022385	Apoptosis, modulate ERK pathway	2.0
Calgranulin B	X06233	Cell cycle progression and differentiation	0.4

### Transcripts of Th17 markers were elevated in tissue lesions during PKDL

**Figure 2:**
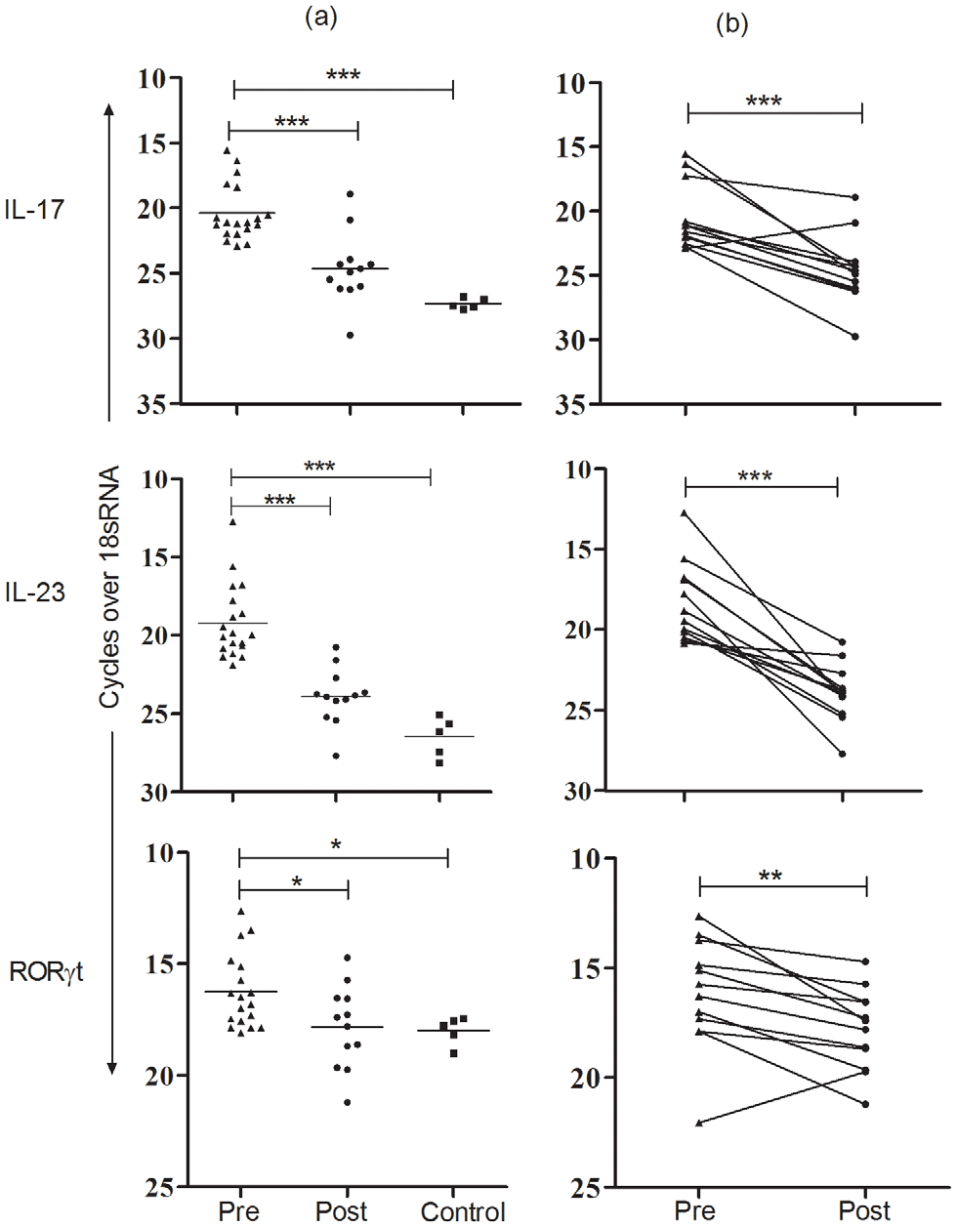
mRNA expression of Th17 markers in PKDL. Relative mRNA levels of IL-17, IL-23 and RORγt was determined by QPCR in (a) tissues lesions at pretreatment (n = 19), post treatment (n = 12), or control tissues (n = 5) or (b) in paired samples (n = 12). The Y axis represents the number of cycles over endogenous control (18sRNA) required to detect the gene expression of interest. *P<0.05, **P<0.01, and ***P<0.001.

On the basis of up regulated expression of IL-17 indicated in array and QPCR analysis, we further investigated gene expression of Th17 markers in PKDL at pre and post-treatment stages. Because IL-17 synthesis requires transcription of RORγt [23], and IL-23 enhances expression of RORγt [24], we assessed the mRNA expression of IL-17, ROR-γt and IL-23 in tissue lesions of PKDL pre-treatment (n = 19), post-treatment stage (n = 12) and healthy individuals (n = 5) using QPCR. Level of Th17 markers was significantly elevated in PKDL compared to post-treatment (IL-17, *P = 0.0003*: IL-23, *P<0.0001*; RORγt, *P* = 0.02) or control (IL-17 and IL-23, *P* = 0.0008; RORγt, *P* = 0.02) (Figure 2a). In follow-up cases (n = 12), chemotherapy significantly curtailed the expression levels (IL-17, *P* = 0.0005; IL-23, *P*<0.0001 & RORγt, *P* = 0.0076) (Figure 2b).

### IL-17^+^ cells were evident in tissue lesions of PKDL patients

**Figure 3:**
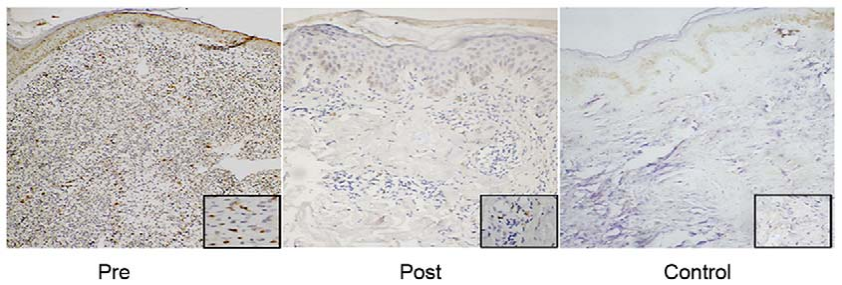
Immuno-histochemical staining of IL-17. A light microscopic analysis of IL-17 immuno-staining in dermal tissue lesion sections of PKDL at pre-treatment, post-treatment stages and normal skin of healthy individuals. Intense IL-17 staining was observed within the inflammatory infiltrate at pre-treatment stage in PKDL. Magnification 10× (inset 40×).

Immunohistochemistry (IHC) was utilized to authenticate translation of IL-17 mRNA into protein in PKDL tissue lesions (n = 8), which showed abundance of IL-17^+^ cells within the dense cellular infiltrate in PKDL compared to control (n = 3). Out of 8 PKDL patients, 5 showed IL17^+^score of 2, 2 showed score of 1, and 1 showed 0 score. After treatment, there was a substantial reduction in IL-17^+^ cells and cell infiltrates with all post-treatment patients showingIL-17 positivity score of 0. In control samples, no IL-17 staining was observed. Representative examples are illustrated in Figure 3.

### Circulatory levels of IL-17 and IL-23 were elevated during PKDL and decreased upon treatment

**Figure 4:**
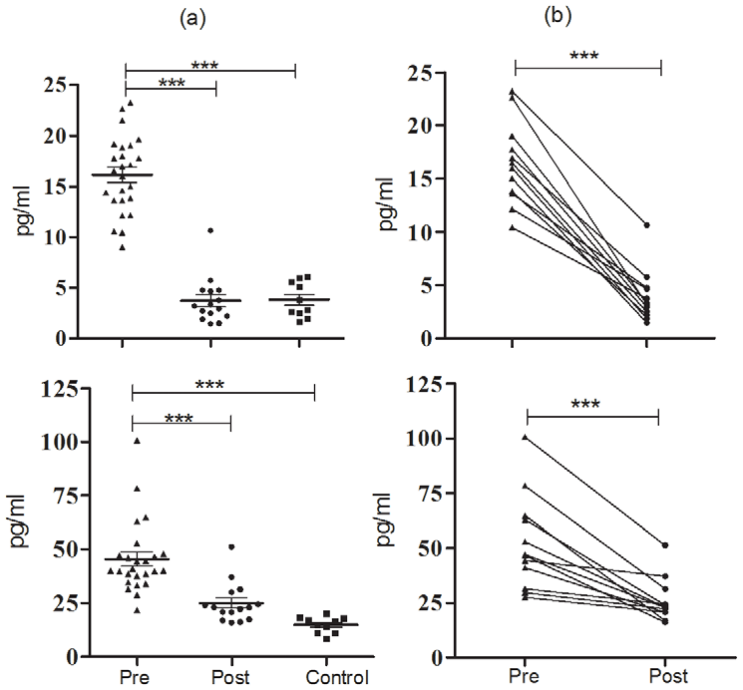
Plasma levels of IL-17 and IL-23 in PKDL. Plasma levels of IL-17 and IL-23 determined by ELISA (a) at pre (n = 25), post-treatment stage (n = 15), controls (n = 10) or (b) in paired samples (n = 12) (b). Individual values (pg/ml) are presented and the mean±SE are shown. **P<0.01, and ***P<0.001.

Circulatory Th17 type responses in PKDL were investigated in plasma samples isolated from blood of PKDL patients at pre-treatment (n = 25), post-treatment stage (n = 12) and healthy individuals (n = 10) using cytokine ELISA. Levels (pg/ml) of cytokines were found to be significantly higher at pre-treatment stage (IL-17: 16.14±0.75, IL-23: 45.48±3.31) compared to post-treatment (IL-17: 3.75±0.59, *P*<0.0001; IL-23: 24.95±2.43, *P*<0.0001) or control (IL-17: 3.828±0.54, *P*<0.0001; IL-23: 14.86±1.17, *P*<0.0001) (Figure 4a). In comparison to pre-treatment levels, a significant down-regulation was observed for IL-17 (16.43±0.99 vs 3.93±0.63; *P*<0.0001) and IL-23 (54.86±4.96 vs 25.90±3.43; *P* = 0.0011) in paired samples (n = 12) (Figure 4b).

### PBMCs of PKDL patients secreted IL-17 and IL-23 in response to Leishmania antigen

**Figure 5:**
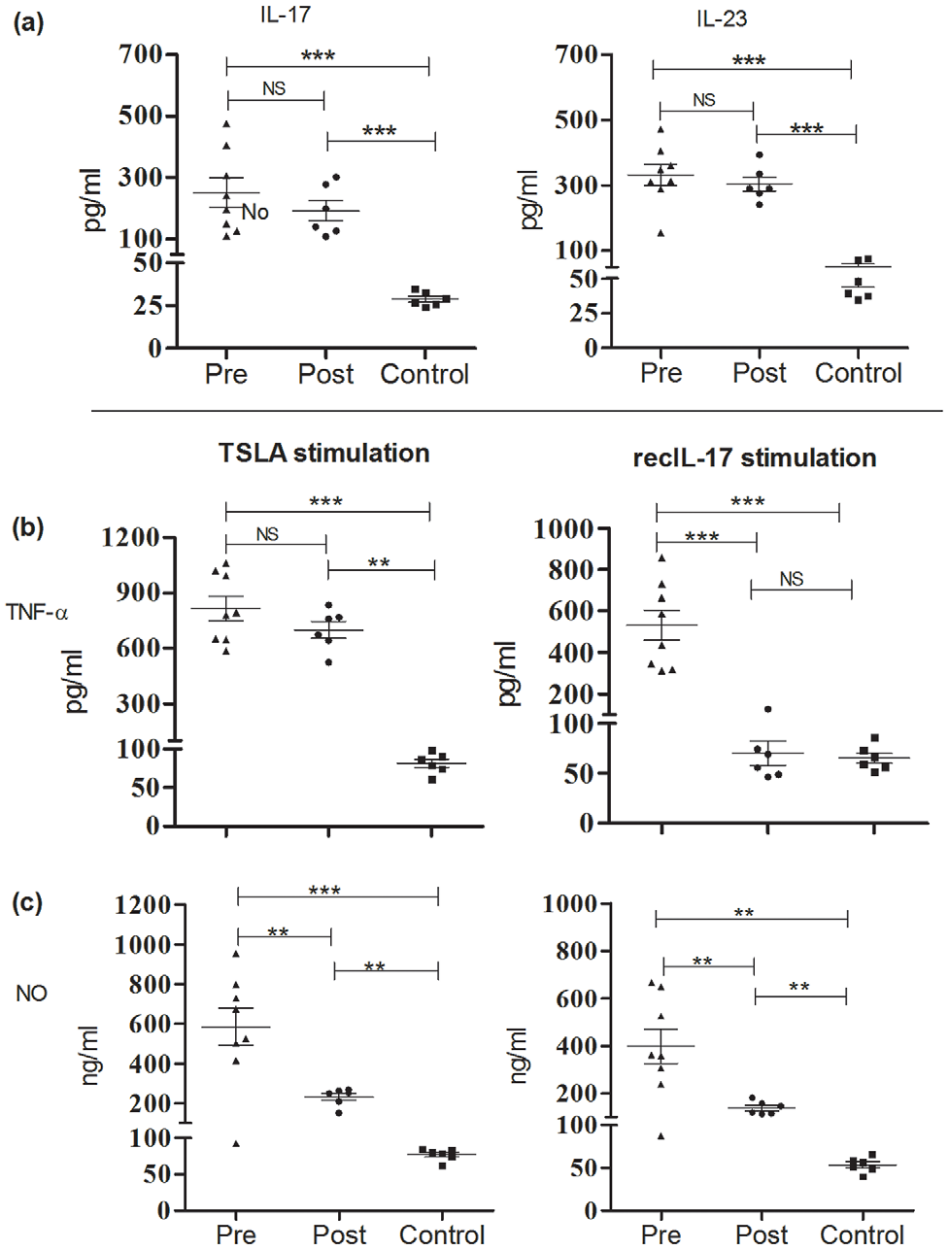
Levels of cytokine nitric oxide and in PBMCs supernatants stimulated with TSLA or recIL-17. (a) IL-17 and IL-23 levels in PBMCs of PKDL pre (n = 8), post (n = 6) and control subjects (n = 6) stimulated with total soluble *Leishmania* antigen (TSLA). (b) Release of TNF-α (pg/ml) or (c) NO (ng/ml) from PBMCs of same set of subjects following incubation with TSLA (10 µg/ml) or recombinant IL-17 (50 ng/ml) for 72 h at 37°C. Cytokine levels were determined by ELISA and NO was quantified by Griess reagent method in culture supernatants. The concentrations shown are the values in the stimulated cultures minus the unstimulated controls. Individual values (pg/ml) are presented and the mean±SE are shown. **P<0.01, and ***P<0.001.

To investigate antigen specific production of Th17 cytokines, PBMCs from PKDL (n = 8) and healthy individuals (n = 6) were cultured and stimulated with TSLA and IL-17 and IL-23 were measured in supernatants of stimulated and unstimulated cells after three days of incubation. Both cytokines (pg/ml) were significantly produced in PKDL (IL-17: 251.1±47.46, IL-23: 331.9±32.78) compared to control (IL-17: 28.93±1.652, *P* = 0.0017; IL-23: 51.15±7.441, P<0.0001) (Figure 5a). The levels in post-treatment samples were comparable to pre treatment stage (IL-17: 191.6±33.43, *P* = 0.35; IL-23: 304.1±21.72, *P* = 0.52) and significantly elevated compared to control (IL-17, *P* = 0.002; IL-23, *P*<0.0001) (Figure 5a).

### IL-17 stimulation enhanced production of TNF-α and NO by PBMCs from PKDL patients

IL-17 is established as a strong inducer of TNF-α production by monocytes [25]. In order to find out the possible association of IL-17 and TNF-α in PKDL, levels of TNF-α (pg/ml) were measured in supernatants from PBMCs of PKDL (n = 8) and healthy individuals (n = 6) stimulated with TSLA or recombinant (rec)IL-17. Both TSLA and recIL-17 stimulated production of TNF-α (815.4±66.01, 530.5±73.68) which was significantly elevated in PKDL compared to control (81.84±5.32, 65.38±5.08, *P* = 0.0007 and 0.0002) (Figure 5b). In response to TSLA, a significant increase was observed in the level of TNF-α in post-treatment samples compared to control (*P* = 0.002, while the levels were comparable between pre and post-treatment stage (699.5±45.39, *P* = 0.28). In response to recIL-17, a significant decrement was observed in the level of TNF-α in post-treatment samples compared to pre-treatment samples (70.16±12.21, *P* = 0.0007). The levels were comparable between post-treatment samples and controls (*P* = 0.81) (Figure 5b). Since IL-17 is an inducer of NO production in various cell types [26], we also assessed the influence of IL-17 on NO production. In PKDL, both TSLA and IL-17 stimulated production of NO (585.7±93.74, 398.9±71.21) which was significantly elevated compared to post-treatment samples (232.1±18.42, *P* = 0.007; 139.1±11.41, *P* = 0.009) or control (76.68±3.23, *P* = 0.0007; 53.26±3.69, *P* = 0.0013) (Figure 5c). The level of NO was significantly higher in post-treatment samples than controls in response to both TSLA and recIL-17 (*P* = 0.002) (Figure 5c).

### Transcripts of TNF-α were positively correlated with IL-17 transcripts

**Figure 6:**
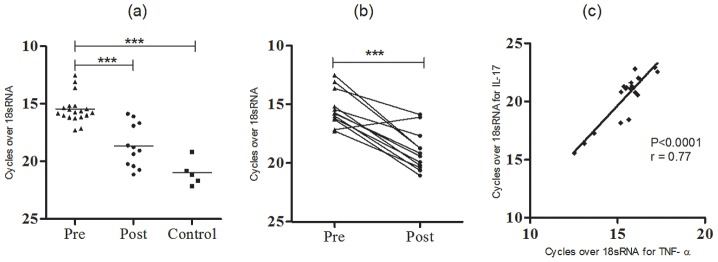
mRNA expression of TNF-α and its correlation with IL-17. Relative mRNA levels of TNF-α in lesion tissues of PKDL patients determined by real time PCR (a) at pretreatment (n = 19) or post treatment (n = 12), or control tissues (n = 5) or (b) in paired samples (n = 12) and (c) Correlation between IL-17 and TNF-α expression in tissue lesions of PKDL. (r) represents Spearman's rank correlation coefficient. **P<0.01, and ***P<0.001.

We measured mRNA expression of TNF-α in PKDL tissue lesions and investigated for any association between TNF-α and IL-17 expressions in the same set of patients. The message levels of TNF-α were found significantly elevated at pre-treatment stage compared to control (P = 0.0008) and decreased with chemotherapy at post-treatment stage (*P*<0.0001). Further analysis established a positive correlation between mRNA expression of IL-17 and TNF-α in tissue lesions of PKDL patients (r = 0.77) (Figure 6). Such correlation was not observed for IL-23 or RORγt.

## Discussion

During the interaction with host cell, parasites attempt to take over cellular functions to their advantage while the host cell counteracts by mounting a variety of defensive responses that include induction of various cytokines, chemokines or other regulatory molecules, all of which are accompanied by changes in gene expression. In the present study, we document the modulation in gene expression profile of various cytokines, chemokines, receptors and apoptotic molecules during *L. donovani* infection in PKDL. The expression of cytokines IFN-γ, TNF-α, IL-10 and TGF-β were found elevated, similar to previous reports [11], with TNF-α showing the highest expression (9 fold) in cytokine category. Further, chemokines including IL-8, CXCL-2, MIP-1α, MIP-1β and MCP-1, were found up regulated, all of which are known to primarily activate the microbicidal activity, by promoting chemotaxis of neutrophils and other target cells. Similarly, in *Leishmania* infection, MCP-1 and MIP-1α have been associated with lesional macrophage infiltration, enhancement of nitric oxide production and stimulation of leishmanicidal activity [27]. Higher expression of chemokines in PKDL implies that these may have a role in shifting the parasite from viscera to dermis, in keeping with a report demonstrating the presence of chemokine binding molecules on the surface of the kinetoplastid parasite [28]. Secondly, as MIP-1α and MIP-1β are potent chemo-attractants for monocyte/macrophages, these may be important in attracting the uninfected immature macrophages. Such cells would provide a safe shelter for the parasite as they get infected but do not kill the parasite, thus contributing towards persistence of parasites.

CD40, a member of the tumor necrosis factor receptor superfamily, interacts with CD40L on activated T cells and helps in immune activation during bacterial, fungal, parasitic and viral infections [29]. In our study, both CD40 and CD40L showed a moderate increment in gene expression in active PKDL. Studies in context with *Leishmania* have demonstrated that the interaction of CD40 with CD40L activates the macrophages resulting in killing of amastigotes [30] but at the same time, a weak CD40 signal promotes IL-10 and reduces IL-12 production [31]. Therefore, it remains a paradox whether CD40 induces host protection or disease promotion.

Another interesting molecule modulated in PKDL was interferon regulatory factor (IRF)-1. It was originally discovered as a protein that binds to DNA sequences termed IRF-Es [32]. It is required for *in vivo* generation of Th1 responses through IL-12 production [33]. Infection of human dendritic cells with *L. major* activated IRF-1 resulting in IL-12 production [34]. Furthermore, IRF-1 acted as a mediator of cytokine-induced apoptosis as hepatocytes from IRF-1-deficient mice were completely resistant to apoptosis induction by IFN-γ [35]. A pronounced expression of IRF-1 pointed towards its involvement in PKDL pathogenesis; however its function during *L.donovani* infection in humans is not known.

In addition to prevalence of Th1/Th2 responses, elevated expression of IL-17 (5 fold) in array results pointed towards the presence of Th17 type responses in PKDL. IL-17 is involved in regulating tissue inflammation, development of autoimmune disorders and in pathogenesis of various infectious diseases [36]. Currently, there is limited information available related to role of IL-17 in *Leishmania* infection. Given the chronic inflammation and neutrophil/macrophage migration during *Leishmania* infection [37], it is logical to speculate that IL-17 may have a pivotal role in PKDL. At pre-treatment stage, elevated levels of IL-17, along with a high expression of Th17 associated markers (IL-23 and RORγt) indicated presence of Th17 type responses. Further, at protein level, immuno-histochemical identification of IL-17^+^ cells in lesional cellular infiltrate and up regulation in both IL-17 and IL-23 levels in circulation of PKDL patients, confirmed lesional as well as systemic presence of Th17 responses during disease. Our results are in conjunction with recent reports on muco-cutaneous Leishmaniasis (ML) and CL, showing elevated levels of IL-17 and associated cytokines [20], [38]. IL-23, a heterodimeric cytokine, is associated with the induction of Th17 cells [39]. Products from microorganisms, including bacteria, intracellular parasites, viruses and fungi are strong inducers of IL-23 production in macrophages, monocytes, neutrophils, and DCs [40]. *M. tuberculosis* infection in human DCs induced IL-23 production which further contributed to IL-17 production from CD4^+^ cells [41]. In this study, IL-23 expression was found up regulated and PBMCs from PKDL patients were able to secrete IL-23 in response to *Leishmania* antigen. In addition to IL-23, generation of human Th17 cells is dependent on IL-1β, TGF-β and IL-6 [19] all of which were found up regulated (5.1, 2.9 and 2.1 fold respectively) in PKDL (Table 2). Thus, we suggest that abundance of IL-17 in PKDL could be due to enhanced IL-23 level which, in association with IL-6, TGF-β and IL-1β, induces differentiation to IL-17 secreting Th17 cells. In follow-up cases, after stimulation with TSLA, the Th17 cytokines levels were comparable to pre treatment stage which may be a memory T cell response. Similar to our results, antigen specific Th17 cytokine responses have been demonstrated in PBMCs from CL and ML patients [20]. In contrast to PKDL, a defective Th17 response in VL against whole parasite or soluble antigen is reported [18]. Possible discrepancy between VL and PKDL could be context dependent due to different niche and clinical manifestation.

To our knowledge, this is the first report demonstrating up regulation of IL-17 during PKDL, which further tended our interest to investigate the role of this cytokine. IL-17 is a known inducer of TNF-α production from endothelial cells, epithelial cells, monocytes and macrophages [25], [42]. Increased production of TNF-α in PBMCs on addition of external IL-17 confirmed the impact of IL-17 towards enhanced TNF-α level. In contrast to PKDL, low abundance of IL-17 has been demonstrated recently in VL [43] that explains the impairment in the production of TNF-α by monocytes in VL [44]. TNF-α is functionally linked to the Th17 pathway by its ability to activate myeloid dendritic cells that synthesize IL-23 and other regulators of T-cell development [45], [46]. Thus, TNF-α may serve as an indirect activator of Th17 responses and a significant positive correlation between these two molecules, as evident in our study, indicated their interrelationship. In addition, IL-17 stimulates NO production in mammalian cells [26] and an elevated level of NO has been shown in PKDL serum samples [47]. Stimulation with IL-17 enhanced the NO level in culture supernatants in PBMCs, establishing the association of IL-17 with elevated NO level. In CL, different studies have shown presence of Th17 responses, up regulation in iNOS expression and increase in NO serum levels [20], [48], [49], however, association between these molecules has not been reported. Here, we demonstrated a direct functional association of IL-17 and NO production during PKDL.

Recently, we have demonstrated accumulation of lesional Tregs and their correlation with parasite load in PKDL [14]. Here we provide evidence for enhanced Th17 responses in the same. Thus, we show a paradoxical gene expression signatures associated with inhibition of the immune response (Tregs) and the proinflammatory response (IL-17, TNF-α) in PKDL. However, no correlation was observed between parasite load and cytokine mRNA levels of Th17 markers. Previously, a number of studies have identified coexistence of Th17 and Treg cells in various diseases [50]–[53]. Considering the association of IL-17 with resistance to *L. donovani* infection [18], the data suggests that during PKDL, parasite induces production of Tregs that counteract inflammatory responses (IL-17, TNF-α and NO) by secreting IL-10 and promoting parasite persistence. It has been shown that adoptive transfer of Tregs inhibits *ex vivo* inflammatory Th17 responses in humans [54], although during active tuberculosis Tregs facilitated mycobacterial replication by inhibiting Th1 rather than Th17 responses [51]. In case of PKDL, further investigations are needed to explore cross talk between these counter acting responses.

Taken together, this study reveals that *L. donovani* infection has a remarkable effect on host cellular gene expression, and identified several new molecules whose implication remains to be defined in context with critical and non-overlapping functions of immuno-modulators in regulating immunity in PKDL. Further, this study demonstrated enhanced expression and antigen specific stimulation of Th17 cytokines and the involvement of IL-17 in PKDL pathogenesis by demonstrating its association with TNF-α and NO production. Based on longitudinal follow-up studies on *L.donovani* infected humans [18] and present results, we suggest that enhanced Th17 responses may have a role in parasite clearance during PKDL. Considering the plasticity between Tregs and Th17 [55], it would be of great interest to investigate reciprocal regulation of these cells in PKDL. Improved mechanistic insight into the collaborative interaction between these immuno-determinants during PKDL would undoubtedly pave way for filling up gaps in our basic knowledge and contribute in developing strategies for disease control.
